# *Candida albicans* as the Sole Organism Cultured from a Perirectal Abscess

**DOI:** 10.1155/2012/913785

**Published:** 2012-09-29

**Authors:** Daniel Tonetti

**Affiliations:** School of Medicine, University of Pittsburgh, S532 Scaife Hall, 3550 Terrace Street, Pittsburgh, PA 15213, USA

## Abstract

Perirectal abscess is a common colorectal condition that may be present with or without a fistula. In most cases where a fistula coexists the organisms cultured are gut-derived organisms whereas skin-derived organisms are more common in patients without fistula formation. *Candida albicans*, despite being an microorganism often found in the gastrointestinal tract, has not previously been reported as an isolate from a perirectal abscess culture. Here we report the case of a patient taking cefazolin in whom a perirectal abscess was diagnosed via computed tomography and aspiration of which demonstrated growth of only *C. albicans*. Prior literature has demonstrated that the microorganisms cultured from patients with perirectal abscesses do not differ between patients in whom antimicrobials had been used previously and those who were antimicrobial-naïve, suggesting that there is a possibility that *C. albicans* is the sole organism responsible for the perirectal abscess in our patient. The patient underwent surgical drainage and was discharged with fluconazole and piperacillin/tazobactam, which led to the satisfactory recovery of the patient.

## 1. Background

Perirectal abscess is a common colorectal disorder, more common in men than women [1, 2], that often requires surgical treatment. Most perirectal abscesses are thought to result from infection that originates in the anal crypts before extending into the anal glands in the intersphincteric plane [3, 4]. Approximately 90% of perirectal abscesses originate as cryptoglandular infections [5], with 80 to 90% of patients also having an accompanying fistula [2, 6]. Clinically, perianal or perirectal pain is the most common presenting symptom in abscesses in this area [7].

In patients with a fistula, the abscesses are more likely to contain multiple different organisms whereas abscesses without fistula tend to have less variety of flora and are more likely to isolate only one organism [2]. This suggests that fistula formation allows for more bacterial inoculation of the abscess. Most aerobic and anaerobic organisms isolated from perirectal abscesses are of gastrointestinal tract and skin flora origin [8]. Skin-derived organisms like *Staphylococcus aureus* are significantly more common in patients without fistula formation [2, 9, 10]. The mechanism of abscess formation from these organisms is thought to be due to skin-derived bacteria blocking apocrine glands, which is supported by the finding that fistulas are less likely to be present with abscesses containing *Staphylococc *[9, 10].

The incidence of gut-derived microorganisms including *E. coli, B. fragilis, *and *Enterococci* cultured from perirectal abscesses has been reported as between 70 and 80%, with these organisms being significantly more prevalent among patients with fistula [2, 9–11]. Abscesses are frequently inoculated with a variety of organisms with most abscesses containing mixed flora, frequently with mixed aerobic-anaerobic flora [2]. Interestingly, diabetic patients with perirectal abscesses may be more susceptible to certain organisms than nondiabetic patients, with *Klebsiella pneumoniae* the most common organism isolated from abscesses in diabetic patients [6]. In contrast the most commonly isolated organism in nondiabetic patients is *Escherichia coli* [6]. 

The dimorphic yeast *Candida albicans* is recognized as an increasingly important human pathogen particularly in the host immunocompromised by advanced age, infection, or immunosuppressive therapy. Candida albicans is often found as a commensal organism in the gastrointestinal tract. There are reports in the literature of *C. albicans* causing abscess in patients who are immunocompromised, diabetic individuals, patients with cancer, or those who are on wide-spectrum antibiotic treatment [12]. There is at least one reported case of a *Candida* spp. abscess in a patient without any of these factors [13]. There have been no previous studies to the best of our knowledge that have identified a fungal organism as a pathogen within a cultured perirectal abscess.

## 2. Case Presentation

 An 86-year-old female patient with a history of hypertension, hyperlipidemia, and lumbar stenosis presented with a complaint of abdominal pain and rectal bleeding. She had just completed a steroid taper for sciatica. Four days prior to admission she stopped having bowel movements and noticed some rectal discomfort with passage of gas and blood per rectum. There was no bleeding one or two days prior to admission, but was brought in by her niece presumably when she learned of these complaints. The patient's appetite had recently been greatly diminished. 

On general exam, the patient was afebrile with a blood pressure of 101/62 and pulse rate of 95/minute. Physical examination revealed internal hemorrhoids with some blood and brown stool in the rectal vault. The patient was stable on the day of admission so she was managed conservatively while investigations were begun. The hematological parameters were as follows: hemoglobin: 13.0 gm/dL, total leukocyte count: 17,000/*μ*L with 83% PMNs (16% bands), platelet count: 249,000/*μ*L, prothrombin time: 15.1 seconds, and INR: 1.2. The biochemical values were fasting blood sugar: 143 mg/dL, sodium: 131 meq/L, potassium: 6.0 meq/L, TSH: 0.438 IU/mL, and creatinine: 0.7 mg/dL.

A colonoscopy was performed on hospital day (HD) 3 which demonstrated moderate rectal prolapse with ulcerated mucosa and a small rectal ulceration. The scope was otherwise unremarkable. On HD 4 the patient had severe overall weakness and fatigue to the point that she was unable to ambulate. This was accompanied by a still-elevated leukocyte count of 16,600/*μ*L with 87% PMNs and 9.5% bands. No rectal bleeding was noted. A urine culture grew coagulase-negative Staphylococcus believed to represent perineal contamination rather than a true urinary tract infection (UTI) pathogen. The patient was treated with cefazolin empirically.

**Figure 1:**
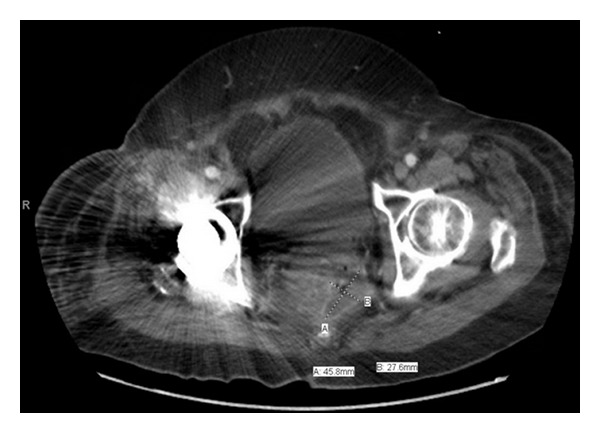
CT scan of the pelvis showing a perirectal fluid collection containing air locules. Fistulous tract to the anorectum not seen in this section.

Because the origin of leukocytosis and accompanying left shift were not readily apparent, computed tomography (CT) of the abdomen and pelvis were performed on HD 9 to evaluate for an occult site of infection. CT demonstrated a 4.6 × 2.8 cm perirectal rim-enhancing fluid collection containing air locules with a fistulous tract to the anorectum (Figure 1), concerning for abscess. Additional findings include rectal wall thickening with perirectal fat stranding concerning proctitis. The patient's antibiotic regimen was switched to piperacillin/tazobactam for anaerobic coverage.

On HD 12, percutaneous drainage of the perirectal abscess revealed 10 mL of cloudy brown liquid. Gram stain demonstrated many polymorphonuclear cells with no microbes. The material was inoculated on Blood agar, MacConkey agar, Chocolate agar, and CNA agar along with several anaerobic agars. The aerobic cultures yielded growth of *Candida albicans* on the second day of culture with no growth on any anaerobic plates. Fluconazole treatment was initiated. The patient was discharged on HD 14 on fluconazole and piperacillin/tazobactam with drain in place which led to satisfactory recovery. 

## 3. Discussion

 Deep *Candida* infections rarely occur in the intact host but their incidence increases greatly in immunosuppressed patients. The incidence of such an infection is also increased in patients who have been treated with cytotoxic drugs, or who are debilitated or following surgery or treatment with broad-spectrum antibiotics. To the best of our knowledge there has not been a reported case of *Candida albicans* within perirectal abscess in the literature. 

*Candida* is known to penetrate the normal gastrointestinal tract and this may be the route of infection in our patient. In a few cases of pancreatic abscesses, cultures yielded pure growth of *Candida albicans*, however all of these patients were receiving broad-spectrum antibiotics which may have inhibited the growth of coinfecting bacteria [14, 15]. It is therefore possible that the abscess in our patient was formed by bacteria, most likely gut-derived given the presence of a fistula, which were eliminated by antibiotics that the patient was taking for several days prior to surgical drainage. 

Surgical drainage is the mainstay of therapy for anorectal abscess with the use of systemic antimicrobial agents indicated in selected cases to prevent bacteremia. There is a slight decrease in recurrence when perirectal abscess is treated with fistulotomy versus drainage alone [16, 17]. Surgical drainage is particularly important because the effectiveness of many antimicrobial agents is mitigated by the abscess capsule, the presence of binding proteins, or deactivating enzymes and the low-pH environment inside the abscess [8]. Interestingly, Toyonaga et al. [2] report that the microorganisms cultured from their array of 514 patients with perirectal abscesses did not differ between patients in whom antimicrobials had been used previously and those who antimicrobial-naïve, which suggests that there is a possibility Candida is the sole organism responsible for the abscess in this patient.
